# PBX3 promotes pentose phosphate pathway and colorectal cancer progression by enhancing G6PD expression

**DOI:** 10.7150/ijbs.86279

**Published:** 2023-08-28

**Authors:** Xinxin Luo, Mankun Wei, Wenfang Li, Hezhao Zhao, Vivi Kasim, Shourong Wu

**Affiliations:** 1 Key Laboratory of Biorheological Science and Technology, Ministry of Education, College of Bioengineering, Chongqing University, Chongqing 400044, China.; 2 The 111 Project Laboratory of Biomechanics and Tissue Repair, College of Bioengineering, Chongqing University, Chongqing 400044, China.; 3 Department of Gastrointestinal Surgery, Chongqing University Cancer Hospital, Chongqing University, Chongqing 400030, China.; 4 Chongqing Key Laboratory of Translational Research for Cancer Metastasis and Individualized Treatment, Chongqing University Cancer Hospital, Chongqing University, Chongqing 400030, China.

**Keywords:** PBX3, G6PD, metabolic reprogramming, pentose phosphate pathway

## Abstract

Metabolic reprogramming is a hallmark of cancers crucial for fulfilling the needs of energy, building blocks, and antioxidants to support tumor cells' rapid proliferation and to cope with the harsh microenvironment. Pre-B-cell leukemia transcription factor 3 (PBX3) is a member of the PBX family whose expression is up-regulated in various tumors, however, whether it is involved in tumor cell metabolic reprogramming remains unclear. Herein, we report that PBX3 is a positive regulator of glucose-6-phosphate dehydrogenase (G6PD), the rate-limiting enzyme in the pentose phosphate pathway (PPP). PBX3 promoted *G6PD* transcriptional activity in tumor cells by binding directly to its promoter, leading to PPP stimulation and enhancing the production of nucleotides and NADPH, a crucial reductant, thereby promoting nucleic acid and lipid biosynthesis while decreasing intracellular reactive oxygen species levels. The PBX3/G6PD axis also promoted tumorigenic potential *in vitro* and *in vivo*. Collectively, these findings reveal a novel function of PBX3 as a regulator of G6PD, linking its oncogenic activity with tumor cell metabolic reprogramming, especially PPP. Furthermore, our results suggested that PBX3 is a potential target for metabolic-based anti-tumor therapeutic strategies.

## Introduction

Metabolic reprogramming is a hallmark of cancers crucial for fulfilling the needs of energy, building blocks, and antioxidants to support tumor cells' rapid growth and to cope with the harsh tumor microenvironment 1. Tumor cells have remarkable flexibility in utilizing energy sources depending on nutrient availability and the heterogeneity of intratumoral cell populations 2. As discovered by Warburg, rapidly proliferating tumor cells consume glucose at an alarming rate compared to normal cells 3, 4 and prefer metabolizing glucose through glycolytic pathways even when oxygen is abundant 5. Aerobic glycolysis improves the adaptability of tumor cells to fluctuating oxygen tension in the harsh tumor microenvironment, converting 85% glucose into lactate to promote tumor migration and invasion, and could suppress anticancer immune effectors 6-8. Tumor cells also utilize glycolytic pathway intermediates for anabolic reactions. Glucose metabolic reprogramming alters the pentose phosphate pathway (PPP), a branch of glycolysis that generates ribose-5-phosphate for high rates *de novo* nucleotide synthesis 9. PPP also generates nicotinamide adenine dinucleotide phosphate (NADPH), a principal intracellular reductant that buffers increased reactive oxygen species (ROS) and supports the synthesis of biological macromolecules such as lipids 10, 11. Although PPP plays a crucial role in tumorigenesis by synthesizing macromolecules and exerting antioxidant functions, the regulatory mechanisms of this pathway have not been fully elucidated.

Pre-B-cell leukemia transcription factor 3 (PBX3) is a transcription factor that belongs to the three amino acid loop extension (TALE) homeobox gene family and contains a highly conserved homologous domain 12, 13. It is a cofactor of homeobox (HOX) proteins and is involved in physiological regulation during embryonic development 14, 15. Moreover, PBX3 is highly expressed in the developing nervous system and adrenal glands, as it controls areas of the medulla oblongata involved in respiration and plays a vital role in regulating steroidogenesis 16, 17. Recent reports have shown the oncogenic role of PBX3 in hematological malignancies and a variety of solid tumors, including acute myeloid leukemia, gastric cancer, colorectal cancer, liver cancer, and cervical cancer 18-22. It is positively correlated with poor prognosis 23 and promotes tumor cell viability by promoting cell cycle progression, cell proliferation, and suppressing apoptosis 24. Furthermore, it could regulate the self-renewal ability of cancer stem cells 25. A previous study also showed that PBX3 is crucial for epithelial-mesenchymal transformation (EMT), thus positively correlated with metastasis and poor prognosis 19. Despite accumulating evidence showing its oncogenic role, whether PBX3 is involved in tumor cell metabolic reprogramming remains unknown.

In this study, to elucidate the role of PBX3 in metabolic reprogramming, we investigated the effects of manipulating PBX3 expression on glucose consumption, lactate production, and the expression of glucose metabolism-related genes. Through *in vitro* and *in vivo* analyses, we revealed the regulatory mechanism of PBX3 on PPP, and subsequently, on tumorigenesis, through transcriptional activation of *glucose-6-phosphate dehydrogenase* (*G6PD*). Together, our study unravels a novel function of PBX3 in regulating tumor metabolic reprogramming.

## Materials and Methods

### Plasmids and constructs

Construction of two shRNA expression vectors targeting different sites of *PBX3* sites were designed according to the algorithm reported previously 24, and the target sites were designed using the algorithm previously reported 26. The sequences are as follow: shPBX3-1: 5′-GGT CAA GGT TTA ATA TTG T-3′; shPBX3-2: 5′-GGG GAA ATG TGA ATA GGC A-3′. Expression vectors for *PBX3* and *G6PD* overexpression vectors (pcPBX3 and pcG6PD, respectively) were constructed as previously described 27, 28.

We utilized the *NheI* and *HindIII* sites of the pGL4.13 vector (Promega, Madison, WI) to insert the -2,137 to +26 and the -1,375 to +26 regions of the *G6PD* promoter for the construction of wild-type G6PD luciferase reporter vectors (G6PD-luc) and G6PD luciferase reporter vector without the predicted PBX3 binding site (G6PD^del^-luc). A Genomic DNA Kit (Tiangen Biotech, Beijing, China) was utilized to extract human genomic DNA from HCT116 cells and use it as a template. PrimeSTAR Max DNA Polymerase (Takara Bio, Dalian, China) was used to amp up the promoter regions. Utilizing the Site-directed Mutagenesis Kit (Beyotime Biotechnology, Shanghai, China), the G6PD luciferase reporter vector with modified PBX3 binding site (G6PD^mut^-luc) was created. *DBD-deleted PBX3* overexpression vector (pcPBX3^DBDdel^) was constructed using pcPBX3 as the template and ClonExpress Ultra One Step Cloning Kit (Vazyme, Nanjing, China).

### Cell lines and cell cultures

The wild-type HCT116, HCC-LM3, and MCF-7 cell lines were obtained from the Cell Bank of the Chinese Academy of Sciences (Shanghai, China) and cultured in McCoy's 5A medium (Gibco, Life Technologies, Grand Island, NY) supplemented with 10% fetal bovine serum (FBS; Biological Industries, Beit Haemek, Israel) and 1% penicillin-streptomycin. The p53-null HCT116 (HCT116^p53null^) cells were generously provided by Dr. Bert Vogelstein at John Hopkins University School of Medicine and maintained in McCoy's 5A medium (Gibco) with 10% FBS (Biological Industries) and 1% penicillin-streptomycin. Mycoplasma Detection Kit-QuickTest (Biotool, Houston, TX) was used to test cell lines periodically for mycoplasma contamination once they had been validated using the short-tandem repetition profiling technique. All cells are cultivated in a humidified incubator at 37 °C with 5% CO_2_. Using Lipofectamine 2000 (Invitrogen Life Technologies, Carlsbad, CA), cells were transfected with the designated vectors in accordance with the manufacturer's instructions.

Cells were seeded in 6-well plates for gene knockdown and overexpression assays, and 2 μg of the appropriate shRNA expression vector or overexpression vector was used to transfect the cells using Lipofectamine 2000. At 24 h post-transfection, puromycin selection was carried out using 1 μg/ml (final concentration) of puromycin for 36 h to remove untransfected cells. To perform rescue experiments, cells were seeded in 6-well plates and transfected with 1 μg shRNA expression vector and 1 μg overexpression vector using Lipofectamine 2000. After 24 h of transfection, the cells were subjected to puromycin selection as previously described to eliminate untransfected cells.

### Animal experiments

To conduct the in *vivo* tumor study, male BALB/*c-nu/nu* mice weighing 18-22 g and aged 6 weeks were obtained from the Chongqing University Cancer Hospital (Chongqing, China). The animal study was approved by the Institutional Ethics Committee of Chongqing University Cancer Hospital and conducted within the premises of the same institution. All animal experiments adhered to the approved Guidelines for the Care and Use of Laboratory Animals at Chongqing University Cancer Hospital, with utmost efforts made to minimize any potential suffering.

To generate an experimental subcutaneous tumor model, BALB/*c-nu/nu* mice were randomized into three groups and experimental subcutaneous tumor models (n = 6) were established with subcutaneous injections showing stable cells (5 × 10^6^ cells/mice). Tumor size (V) was evaluated by a caliper every two days using the following equation: V = a × b^2^/2; where a and b are the major and minor axes of the tumor, respectively. The researcher was blinded to group allocation during the evaluation.

### Clinical human colon carcinoma specimen

Samples of human colon carcinoma were obtained from patients with colon carcinoma undergoing surgical procedures at Chongqing University Cancer Hospital (Chongqing, China) and stored in the Biological Specimen Bank of Chongqing University Cancer Hospital. No patients received chemotherapy, radiation, or other adjuvant therapies before surgery. Samples were snap-frozen in liquid nitrogen. Prior patient's written informed consent was obtained. Ethics approval for the experiments was obtained from the Institutional Research Ethics Committee of Chongqing University Cancer Hospital and the experiments were conducted in accordance with the the Declaration of Helsinki.

### Western blotting and quantitative reverse transcription-PCR (qRT-PCR) analysis

Detailed methods for performing western blotting and qRT-PCR analysis are described in the Supplementary Materials and Methods. The sequences of the primers and antibodies used are shown in Supplementary Tables S1 and S2, respectively.

### Measurements of glucose consumption, lactate production, G6PD enzymatic activity, and intracellular NADPH level

Cells were transfected with the indicated shRNA expression vector or overexpression vector and selected using puromycin as previously described. The amount of glucose consumed and the amount of lactate produced in the culture medium were analyzed using the Glucose Colorimetric Assay Kit (BioVision, Milpitas, USA) and the Lactate Assay Kit (KeyGen Biotech, Jiangsu, China), respectively, according to the manufacturer's instructions. The enzymatic activity of G6PD and intracellular NADPH/NADP^+^ levels were detected using the G6PD Assay Kit (Yuanye, Shanghai, China) and AmpliteTM Colorimetric NADPH/NADP^+^ Ratio Assay Kit (Comin Bio, Suzhou, China), respectively, according to the manufacturer's instructions. The total amount of protein determined using the BCA Protein Assay Kit (Beyotime Biotechnology) was used for normalization of the results obtained.

### Intracellular ROS level

Cells were transfected with either the shRNA expression vector indicated or the overexpression vector and selected using puromycin as previously described. Twenty-four hours after re-seeding in 6-well plates, the cells were harvested and stained with DCFH-DA using the Reactive Oxygen Assay Kit (Beyotime Biotechnology). Next, we measured intracellular ROS levels using flow cytometry.

### Statistical analysis

All quantification results were presented as mean ± S.D. (n = 3; unless otherwise indicated). Statistical analysis was performed using unpaired two-tailed Student's t test performed using GraphPad Prism 8.0 software. Statistical analysis was conducted using one-way ANOVA for clinical specimens and xenograft experiments. Values of *P < 0.05 were considered to indicate statistical significance.

## Results

### PBX3 regulates glucose metabolism

**Figure 1:**
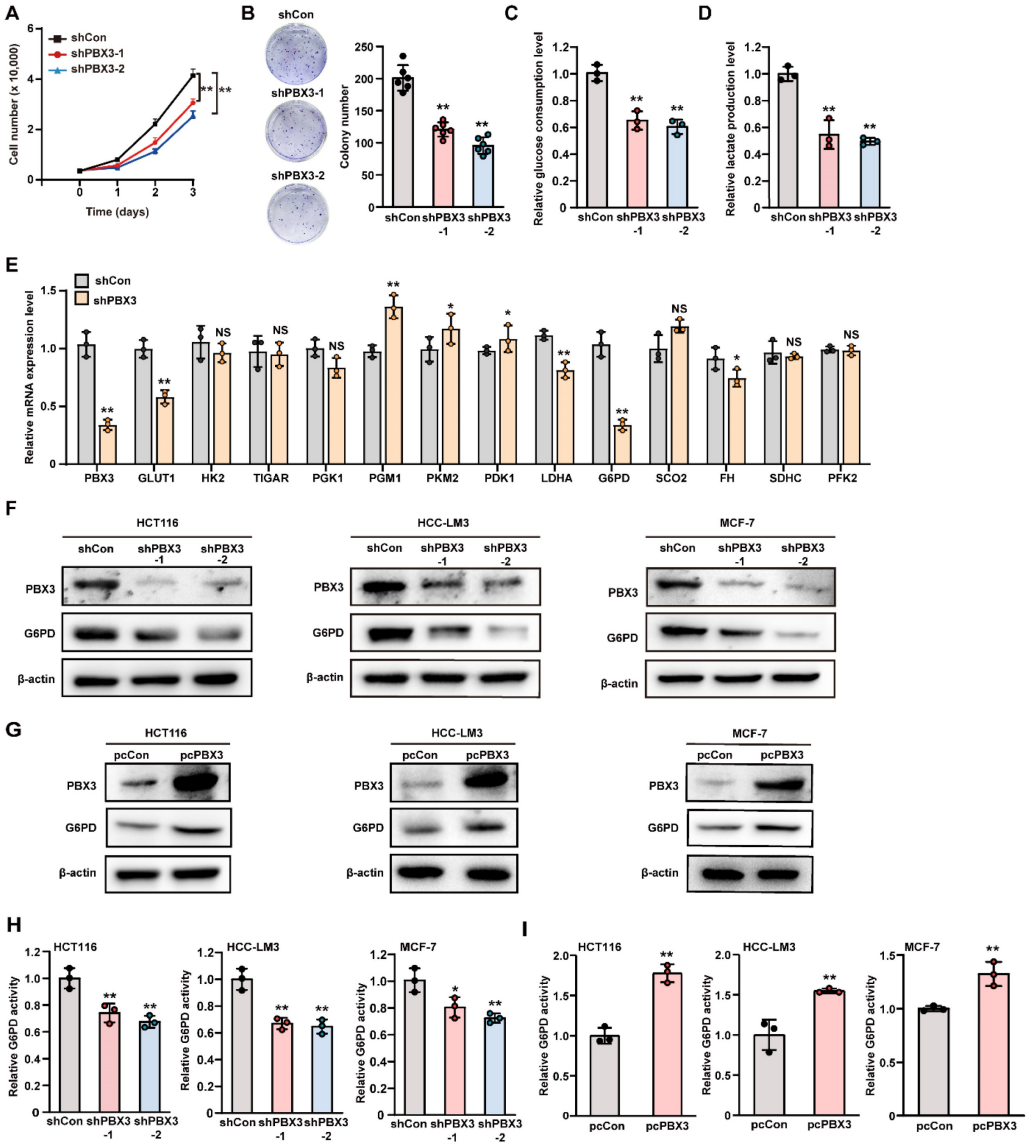
** PBX3 regulates glucose metabolism. A.** Viability of *PBX3*-knocked down HCT116 cells. **B.** Colony formation potential of *PBX3*-knocked down HCT116 cells. Representative images (left) and quantification results (right; n = 6) are shown. **C-D.** Glucose consumption (C) and lactate production (D) levels in *PBX3*-knocked down HCT116 cells. **E.** mRNA expression levels of glucose metabolism-related genes in *PBX3*-knocked down HCT116 cells, as analyzed using qRT-PCR. **F-G.** G6PD protein expression levels in *PBX3*-knocked down (F) and *PBX3*-overexpressed (G) tumor cells, as examined using western blotting. **H-I.** G6PD activity in *PBX3*-knocked down (H) and *PBX3*-overexpressed (I) tumor cells**.** Cells transfected with shCon or pcCon were used as control. β-actin was used for qRT-PCR normalization and as western blotting loading control. Total protein was used for normalizing the levels of glucose consumption, lactate production, and G6PD activity. Quantification data are shown as mean ± SD (n = 3; unless otherwise indicated). pcCon: pcEF9-Puro; *P < 0.05; **P < 0.01; NS: not significant.

To investigate the role of PBX3 in glucose metabolism in tumor cells, we first analyzed the effect of knocking down *PBX3* on tumor cell viability and colony formation potentials using two shRNA expression vectors targeting different sites of *PBX3* **(Supplementary Figure S1A, B).** Knocking down *PBX3* significantly suppressed HCT116 cell viability as well as their colony formation potential **(Figure 1A, B)**. We next examined the effect of knocking down *PBX3* on tumor cell glucose consumption rate and lactate production levels. The results clearly showed that knocking down *PBX3* suppressed the glucose consumption rate and lactate production **(Figure 1C, D).** Meanwhile, *PBX3* overexpression robustly increased them **(Supplementary Figure S1C-E)**. Together, these results indicate that PBX3 might be involved in tumor cell glucose metabolism.

Next, to elucidate the underlying molecular mechanism, we investigated the effect of PBX3 on the expression of genes associated with glucose metabolism. As shown in **Figure 1E**, among the genes whose mRNA expression were affected by* PBX3* knockdown, G6PD showed the most significant alteration. The positive regulation of PBX3 on G6PD mRNA expression was further validated using two shRNA expression vectors targeting different sites of *PBX3* **(Supplementary Figure S2A)** and a *PBX3* overexpression vector **(Supplementary Figure S2B)**. We further confirmed the effect of PBX3 on G6PD protein level and found that knocking down *PBX3* robustly suppressed the protein level of G6PD in colon cancer cells HCT116, hepatocellular carcinoma cells HCC-LM3, and breast cancer cells MCF-7 **(Figure 1F)**, while overexpressing *PBX3* conspicuously increased G6PD protein levels in these cells **(Figure 1G)**. Concomitantly, PBX3 alteration positively regulated G6PD enzymatic activity in HCT116, HCC-LM3, and MCF-7 cells **(Figure 1H, I)**. Together, these results suggest that PBX3 is a positive regulator of G6PD and is crucial for glucose metabolic reprogramming in tumor cells.

### PBX3 promotes PPP in tumor cells

**Figure 2:**
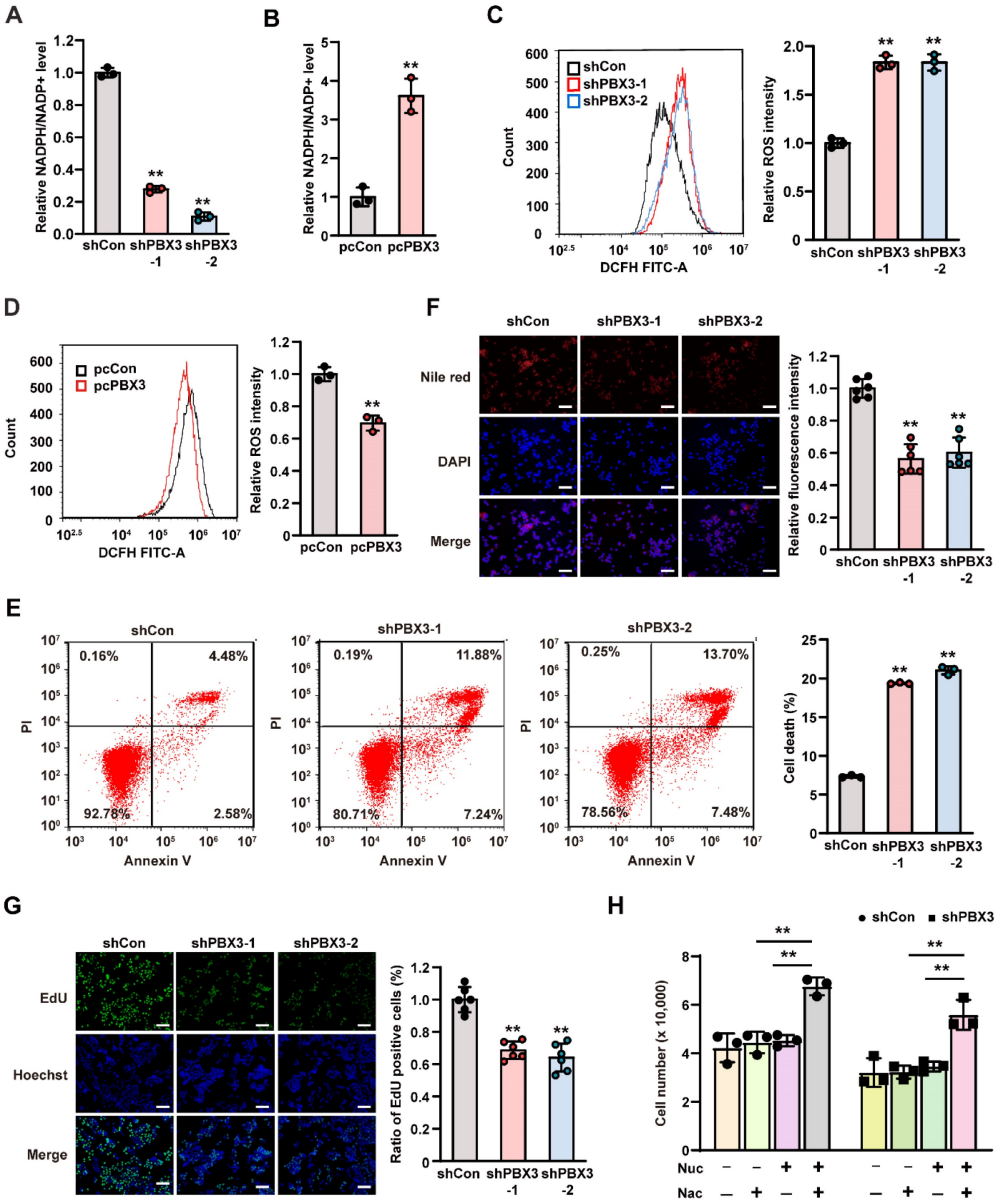
** PBX3 promotes tumor cells PPP. A-B.** Intracellular NADPH/NADP^+^ ratio in *PBX3*-knocked down (A) and *PBX3*-overexpressed (B) HCT116 cells **C-D.** Intracellular ROS level in *PBX3*-knocked down (C) and *PBX3*-overexpressed (D) HCT116 cells*.* Representative images (left) and quantification results (right; n = 3) are shown.** E.** Percentage of apoptotic cells in *PBX3*-knocked down HCT116 cells, as examined using Annexin V/PI staining and flow cytometry.** F.** Lipid accumulation in *PBX3*-knocked down HCT116 cells, as examined using Nile red staining. Representative images (left) and quantification results (right; n = 6) are shown. **G.** Proliferation potential of *PBX3*-knocked down HCT116 cells, as examined using EdU-incorporation assay. Representative images (left) and quantification results (right; n = 6) are shown. **H.** Viability of *PBX3*-knocked down HCT116 cells cultured in the presence of nucleosides mixture (Nuc) and/or N-acetyl-L-cysteine (Nac), as measured on the third day after the addition of Nuc and/or Nac (n = 3). Cells transfected with shCon or pcCon were used as control. β-actin was used as western blotting loading control. Total protein was used for normalizing NADPH/NADP^+^ level. Scale bars: 200 μm. Quantification data are shown as mean ± SD (n = 3; unless otherwise indicated); pcCon: pcEF9-Puro; **P < 0.01.

PPP consists of an oxidizing branch and a non-oxidizing branch, with G6PD as its first rate-limiting enzyme 29. In tumor cells, PPP directs glucose flux to its oxidative branches and produces NADPH, a cellular reductant that can act as an ROS scavenger and thereby is essential for tumor cell antioxidant defense 30. To elucidate the role of PBX3 in PPP, we examined the effect of PBX3 expression on intracellular NADPH levels. We found that knocking down *PBX3* significantly reduced cellular NADPH levels while enhancing its oxidative form, NADP^+^** (Supplementary Figure S3A, B)**, resulting in the decrease of the NADPH/NADP^+^ ratio **(Figure 2A)**. These effects were reversed by *PBX3* overexpression **(Figure 2B, Supplementary Figure S3C, D)**. Concomitantly, knocking down *PBX3* robustly increased intracellular ROS level, while *PBX3* overexpression had the opposite effect **(Figure 2C, D)**. Furthermore, *PBX3* knockdown also robustly increased the percentage of apoptotic cells **(Figure 2E)**.

NADPH plays a crucial role in lipid synthesis by functioning as an anabolic hydrogen donor involved in lipid synthesis as well as in the synthesis of fatty acids and cholesterol from acetyl CoA 31. Hence, we next evaluated the effect of PBX3 on lipid accumulation. Nile red staining results showed that lipid accumulation was clearly suppressed in *PBX3*-knocked down HCT116 cells, and increased in *PBX3*-overexpressed HCT116 cells **(Figure 2F and Supplementary Figure S4)**. These results were in accordance with the positive regulation of PBX3 on cellular NADPH levels.

Furthermore, PPP is also the main source of ribose-5-phosphate, the building blocks of nucleotides, which serve as substrates for continuous DNA replication. Indeed, knocking down *PBX3* could significantly inhibit intracellular DNA replication, as indicated by the decrease of EdU-positive cells** (Figure 2G)**. Together, these results indicated that knocking down *PBX3* increased intracellular ROS and apoptosis while suppressing nucleotide biosynthesis. Hence, we attempted to rescue the viability of *PBX3*-knocked down HCT116 cells by adding N-acetyl-L-cysteine, an ROS scavenger, and the nucleosides (4 ribonucleosides and 4 deoxyribonucleosides) required for DNA and RNA synthesis. The addition of N-acetyl-L-cysteine or nucleosides alone did not significantly affect the viability of *PBX3*-knocked down HCT116 cells, however, the addition of both clearly restored cell viability (**Figure 2H**). Together, these results suggested that PBX3 positively regulates PPP in tumor cells, thereby promoting their viability.

### G6PD is essential for PBX3-induced PPP

**Figure 3:**
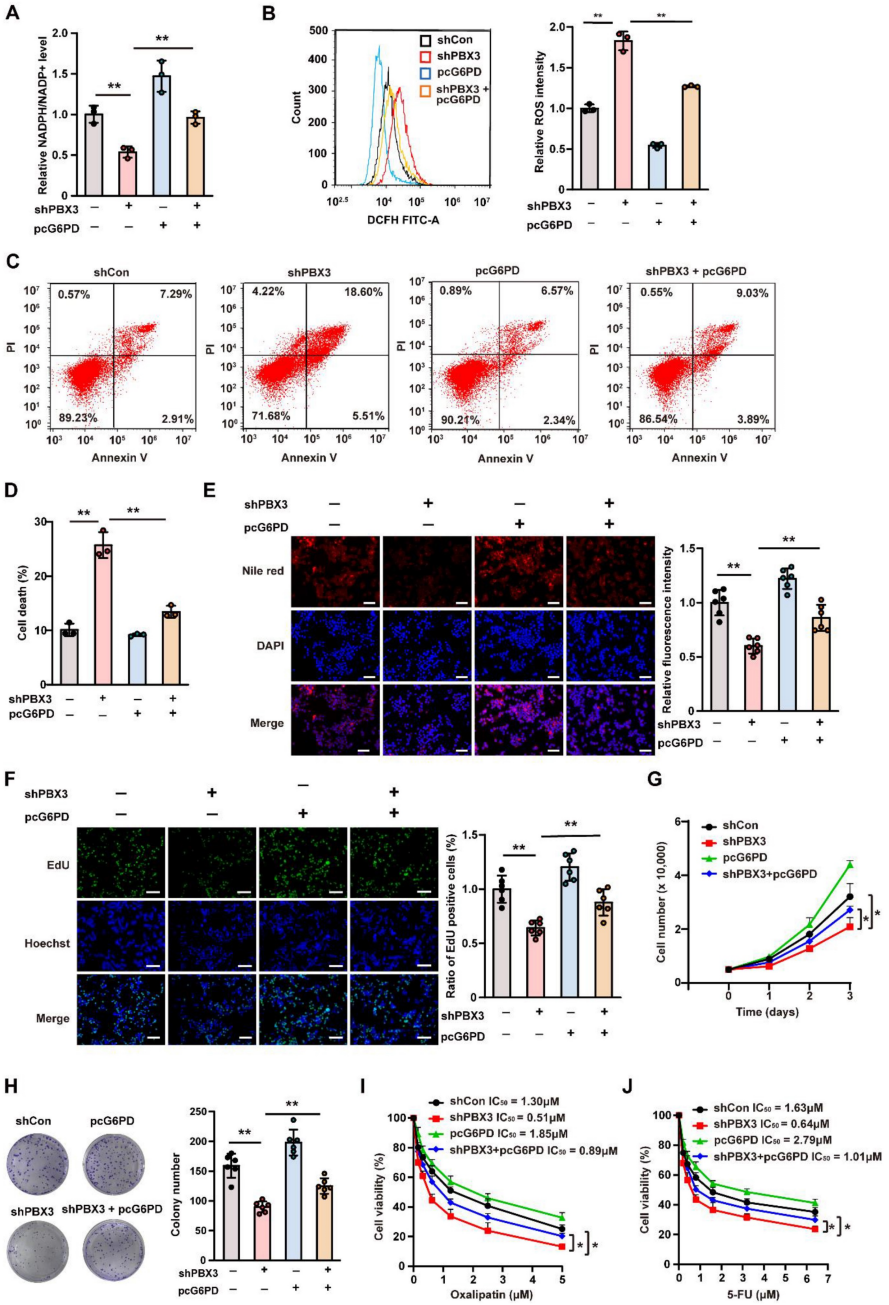
** G6PD is essential for PBX3-induced PPP. A.** Intracellular NADPH/NADP^+^ ratio in *PBX3*-knocked down, *G6PD*-overexpressed HCT116 cells.** B.** Intracellular ROS level in *PBX3*-knocked down, *G6PD*-overexpressed HCT116 cells. Representative images (left) and quantification results (right; n = 3) are shown. **C-D.** Percentage of apoptotic cells in *PBX3*-knocked down, *G6PD*-overexpressed HCT116 cells, as examined using Annexin V/PI staining and flow cytometry. Representative images (C) and quantification results (D) are shown**. E.** Lipid accumulation in *PBX3*-knocked down, *G6PD*-overexpressed HCT116 cells, as examined using Nile red staining. Representative images (left) and quantification results (right; n = 6) are shown. **F.** Proliferation potential of *PBX3*-knocked down, *G6PD*-overexpressed HCT116 cells, as examined using EdU-incorporation assay. Representative images (left) and quantification results (right; n = 6) are shown. **G.** Viability of *PBX3*-knocked down, *G6PD*-overexpressed HCT116 cells at indicated time-points. **H.** Colony formation potential of *PBX3*-knocked down, *G6PD*-overexpressed HCT116 cells. Representative images (left) and quantification results (right; n = 6) are shown. **I-J.** Viabilities of *PBX3*-knocked down, *G6PD*-overexpressed HCT116 cells treated with indicated doses of oxaliplatin (I) and 5-FU (J). Cells transfected with shCon and/or pcCon were used as control. β-actin was used as western blotting loading control. Total protein was used for normalizing NADPH/NADP^+^ level. Scale bars: 200 μm. Quantification data are shown as mean ± SD (n = 3; unless otherwise indicated); pcCon: pcEF9-Puro; *P < 0.05, **P < 0.01.

To confirm whether PBX3 regulates PPP in tumor cells through G6PD, G6PD expression and activity were rescued in *PBX3*-knocked down HCT116 cells by overexpression **(Supplementary Figure S5A, B)**. *PBX3* overexpression significantly restored cellular NADPH level and ameliorated the increase in NADP^+^ in *PBX3*-knocked down HCT116 cells **(Supplementary Figure S5C, D)**, thereby restoring the NADPH/NADP^+^ ratio** (Figure 3A)**. Concomitantly, *G6PD* overexpression prevented cellular ROS increase and apoptosis mediated by *PBX3* knockdown **(Figure 3B-D)**. Furthermore, it restored lipid accumulation **(Figure 3E)** and increased the number of EdU-positive cells **(Figure 3F)** suppressed by *PBX3* knockdown, indicating that *G6PD* overexpression could compensate for the defect in biomacromolecule synthesis caused decreased PBX3. Subsequently, *G6PD* overexpression prevented the suppressive effect of *PBX3* knockdown on the viability and colony formation potential of HCT116 cells **(Figure 3G, H)**.

Furthermore, PPP is also critical for tumor drug resistance as it could promote NADPH to scavenge excessive ROS induced by DNA damage-based antitumor drugs 32. Hence, we further investigated the effect of the PBX3/G6PD axis on tumor cell resistance against 5-FU and oxaliplatin, which induce DNA damage by producing excessive ROS 33, 34. Knocking down *PBX3* increased the suppressive effects of 5-FU and oxaliplatin on HCT116 cells viability at every drug concentration tested, while *G6PD* overexpression abolished these effects (**Figure 3I, J**). The decreased IC_50_ values also supported that knocking down *PBX3* sensitized HCT116 cells to 5-FU and oxaliplatin, which was prevented by overexpressing *G6PD.* Together, these results confirm that G6PD is crucial for PBX3 regulation of tumor cell PPP.

### PBX3 alters PPP in a p53-independent manner

**Figure 4:**
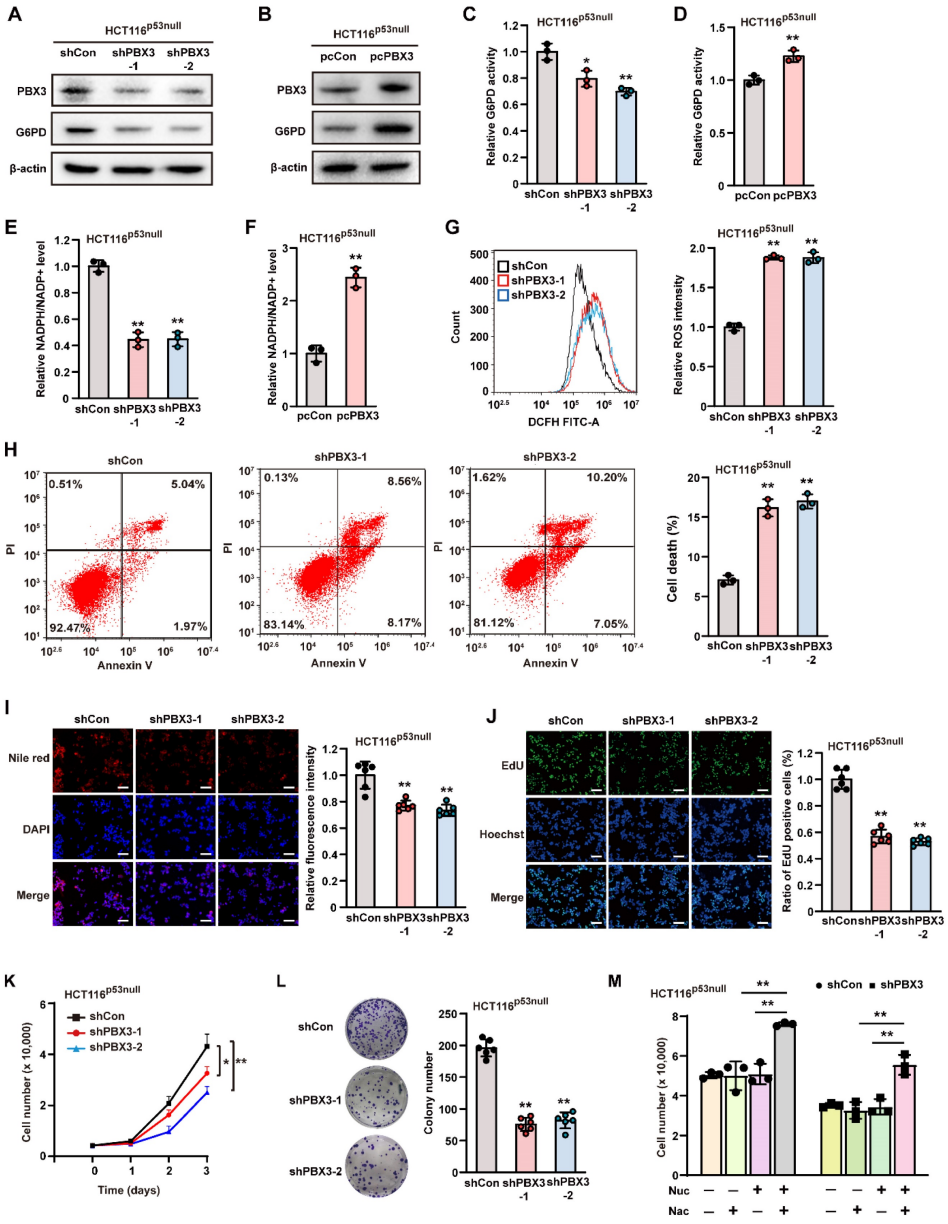
** PBX3 enhances PPP in a p53-independent manner. A-B.** G6PD protein expression level in* PBX3*-knocked down (A) and *PBX3*-overexpressed (B) HCT116^p53null^ cells, as determined using western blotting. **C-D.** G6PD enzymatic activity in *PBX3*-knocked down (C) and *PBX3*-overexpressed (D) HCT116^p53null^ cells**. E-F.** Intracellular NADPH/NADP^+^ ratio in *PBX3*-knocked down (E) and* PBX3*-overexpressed (F) HCT116^p53null^ cells.** G.** Intracellular ROS level in *PBX3*-knocked down HCT116^p53null^ cells. Representative images (left) and quantification results (right) are shown.** H.** Percentage of apoptotic cells in *PBX3*-knocked down HCT116^p53null^ cells, as examined using Annexin V/PI staining and flow cytometry. **I.** Lipid accumulation in *PBX3*-knocked down HCT116^p53null^ cells, as examined using Nile red staining. Representative images (left) and quantification results (right; n = 6) are shown. **J.** Proliferation potential of *PBX3*-knocked down HCT116^p53null^ cells, as examined using EdU-incorporation assay. Representative images (left) and quantification results (right; n = 6) are shown. **K-L.** Viability (K) and colony formation potential (L; n = 6) of *PBX3*-knocked down HCT116^p53null^ cells. **M.** Viability of *PBX3*-knocked down HCT116^p53null^ cells cultured in the presence of Nuc and/or Nac, as measured on the third day after the addition of Nuc and/or Nac. Cells transfected with shCon or pcCon were used as control. β-actin was used as western blotting loading control. Total protein was used for normalizing G6PD enzymatic activity and NADPH/NADP^+^ level. Scale bars: 200 μm. Quantification data are shown as mean ± SD (n = 3; unless otherwise indicated). pcCon: pcEF9-Puro; *P < 0.05; **P < 0.01.

Previous studies have shown that p53 is a critical inhibitor of G6PD enzymatic activity 11, 35; meanwhile, we previously found that PBX3 could inhibit *p53* transcription 24. Hence, we next explored whether PBX3 regulation of G6PD occurs in a p53-dependent manner using *p53-null* HCT116 (HCT116^p53null^) cells **(Supplementary Figure S6A)** and examined the effect of knocking down *PBX3* on G6PD expression. Our results showed that *PBX3* knockdown led to a significant decrease in G6PD mRNA **(Supplementary Figure S6B)**, while *PBX3* overexpression increased it **(Supplementary Figure S6C)**. Furthermore, G6PD protein expression levels **(Figure 4A, B)** as well as its enzymatic activities **(Figure 4C, D)** also showed similar tendencies.

Next, we investigated whether PBX3 could induce PPP in the absence of *p53*. Knocking down *PBX3* also reduced cellular NADPH level and increased that of NADP^+^ **(Supplementary Figure S7A, B)**, leading to a significant decrease in the NADPH/NADP^+^ ratio in HCT116^p53null^ cells **(Figure 4E)**. Concomitantly, *PBX3* overexpression increased cellular NADPH while decreasing NADP^+^ **(Supplementary Figure S7C, D)**, thereby enhancing the NADPH/NADP^+^ ratio **(Figure 4F)**. Furthermore, knocking down *PBX3* significantly elevated intracellular ROS levels and apoptosis in HCT116^p53null^ cells **(Figure 4G, H)**.

Next, we analyzed whether PBX3 could affect lipid accumulation and DNA replication in the absence of *p53*. Knocking down *PBX3* clearly reduced lipid accumulation** (Figure 4I)** and blocked DNA replication **(Figure 4J)** in HCT116^p53null^ cells. Furthermore, knocking down *PBX3* suppressed the viability and colony formation potential of HCT116^p53null^ cells **(Figure 4K, L)**. The addition of N-acetyl-L-cysteine and a nucleoside mixture restored the viability of *PBX3*-knocked down HCT116^p53null^ cells **(Figure 4M)**. Together, these results indicate that PBX3 could activate PPP in tumor cells in the absence of p53.

### PBX3 binds to the G6PD promoter and promotes its transcription

**Figure 5:**
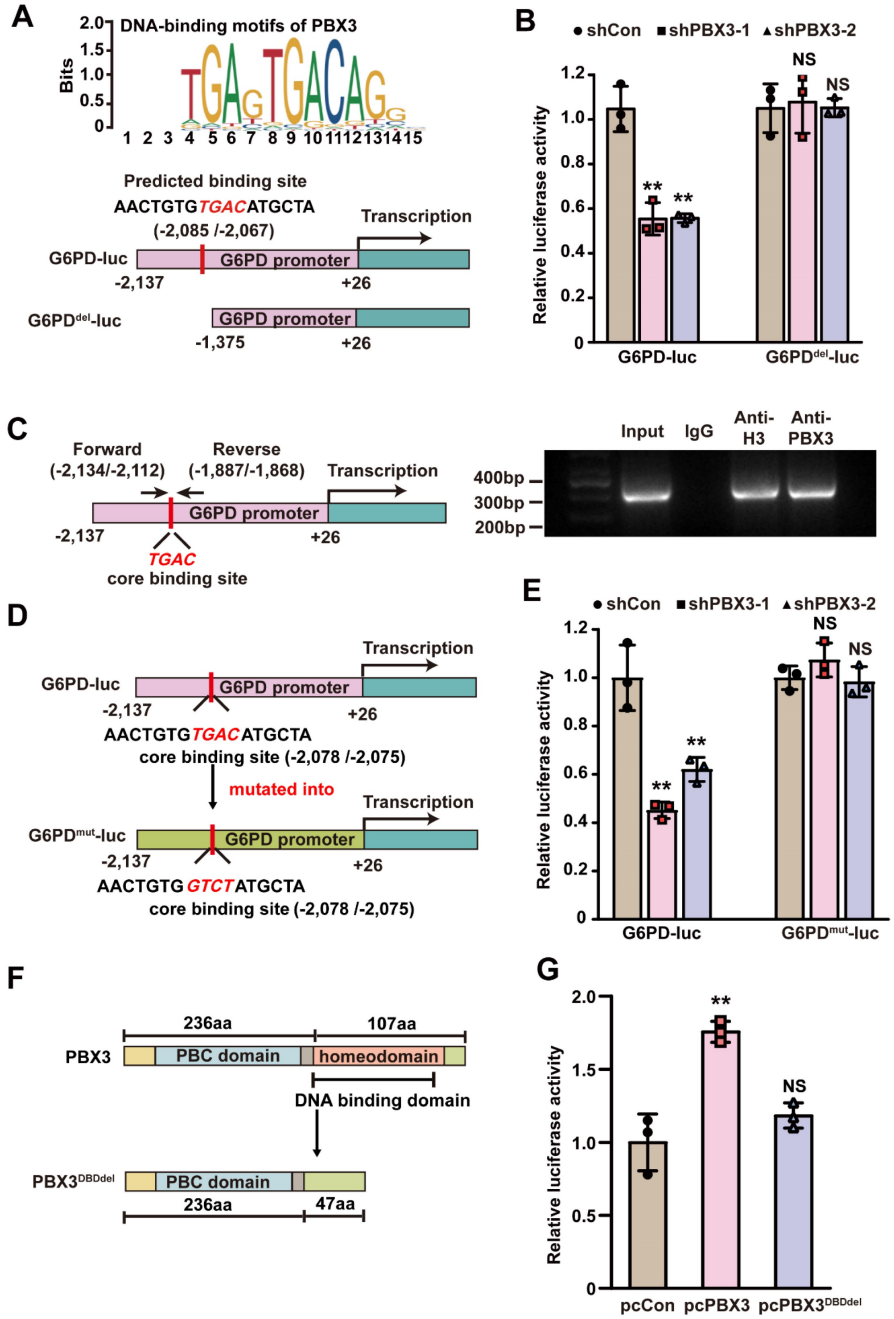
** PBX3 binds to the *G6PD* promoter and promotes its transcription. A.** Schematic diagram of DNA-binding motif of PBX3 as predicted using JASPAR (upper panel) and *G6PD* promoter reporter vectors with or without predicted PBX3 binding site (G6PD-luc and G6PD^del^-luc, respectively; lower panels). **B.** Relative luciferase activities of G6PD-luc and G6PD^del^-luc in *PBX3*-knocked down HCT116 cells. **C.** Binding capacity of PBX3 to the predicted region in *G6PD* promoter, as examined using ChIP assay with an anti-PBX3 antibody followed by PCR. The predicted PBX3 binding site in the promoter region of G6PD and the location of the primer set used for PCR are shown. Anti-histone H3 antibody was used as a positive control. **D-E.** Relative luciferase activities of G6PD-luc and G6PD^mut^-luc in *PBX3*-knocked down HCT116 cells. Schematic diagram (D) and relative luciferase activities to control (E) are shown. **F-G.** Relative luciferase activities of G6PD-luc in HCT116 cells overexpressing *PBX3* or *PBX3^DBDdel^*. Schematic diagram of PBX3^DBDdel^ (F) and relative luciferase activities (G) are shown. Cells transfected with shCon or pcCon were used as a control. Quantification data are shown as mean ± SD (n = 3). pcCon: pcEF9-Puro; **P < 0.01; NS: not significant.

To elucidate the molecular mechanism of PBX3-mediated regulation of G6PD, we predicted the presence of a PBX3 binding motif on the *G6PD* promoter using JASPAR (http://jaspar.genereg.net) 27 and identified a predicted PBX3 binding site in the -2,085 to -2,067 region of the *G6PD* promoter. Therefore, we constructed luciferase reporter assay vectors with (G6PD-luc) and without (G6PD^del^-luc) the predicted binding site **(Figure 5A)**. Luciferase reporter assay results showed that* PBX3* knockdown could suppress G6PD-luc activity but not that of G6PD^del^-luc** (Figure 5B)**. These results indicated that the -2,137 to -1,376 region is essential for PBX3 regulation of *G6PD* promoter activity.

Next, we analyzed whether PBX3 binds to the *G6PD* promoter at the predicted site. To this end, we performed a ChIP assay utilizing a set of primers flanking the predicted PBX3 binding site on the *G6PD* promoter, and detected the fragment of the -2,134 to -1,868 region of the *G6PD* promoter in the chromatin immunoprecipitated using the anti-PBX3 antibody. This indicates that PBX3 could bind to the -2,134 to -1,868 region of the *G6PD* promoter, which includes the predicted PBX3 binding site **(Figure 5C)**. Finally, to assess whether the predicted binding site was functional, a G6PD luciferase reporter vector was constructed with four point mutations in the PBX3 core binding site (G6PD^mut^-luc): the TGAC sequence in the wild-type *G6PD* promoter was mutated into GTCT (**Figure 5D**). The results indicated that while knocking down *PBX3* suppressed the luciferase activity of the wild-type G6PD-luc reporter, it had no significant effect on the G6PD^mut^-luc reporter. **(Figure 5E)**.

To further confirm the direct PBX3 regulation of *G6PD* transcription, the DNA-binding domain (DBD) of PBX3 was predicted using UniProt (https://www.uniprot.org/) to construct a DBD-deleted *PBX3* overexpression vector (pcPBX3^DBDdel^; **Figure 5F**). As shown by the luciferase reporter assay results in **Figure 5G**, while overexpressing *PBX3* could significantly promote G6PD-luc activity, overexpressing *PBX3^DBDdel^* had no significant effect. Together, these results indicate that PBX3 could directly bind to the *G6PD* promoter, most plausibly through its predicted binding site in the -2,085 to -2,067 region of the *G6PD* promoter, and that such binding is critical for *G6PD* transcriptional regulation.

### PBX3/G6PD axis regulates the tumorigenic potential of CRC cells

**Figure 6:**
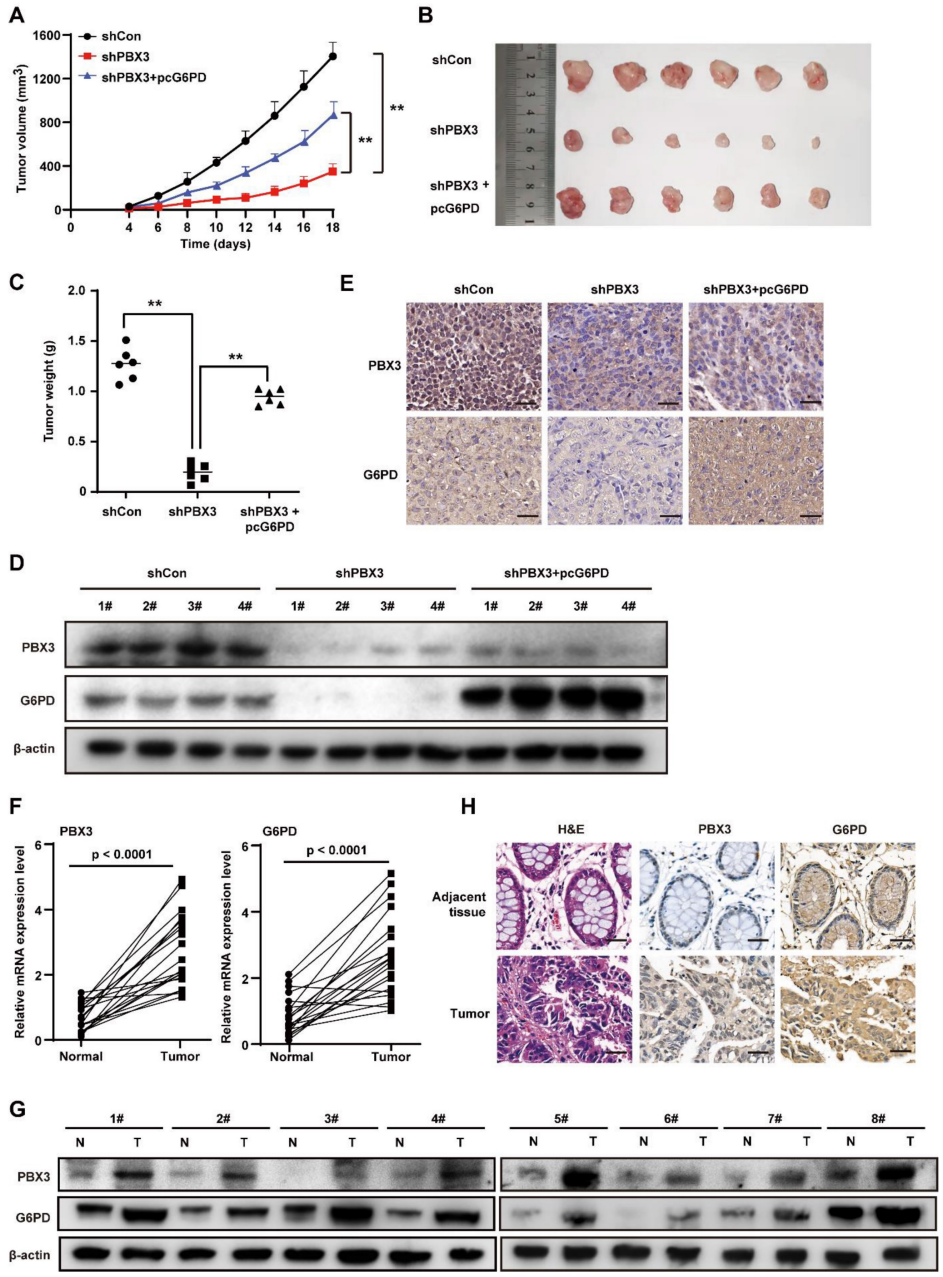
** PBX3/G6PD axis regulates the tumorigenic potential of CRC cells. A-C.** Tumorigenic potentials of shCon+pcCon (shCon), shPBX3+pcCon (shPBX3), and shPBX3+pcG6PD (shPBX3+pcG6PD) stable cell lines were examined* in vivo* by subcutaneous injection of these cells into Balb/*c-nu/nu* mice (n = 6). Tumor volume at indicated time points (A), morphological images at 18 days after transplantation (B), and tumor weight at 18 days after transplantation (C) are shown. **D-E.** PBX3 and G6PD protein expression levels in the xenografted tumors, as examined using western blotting (D) and immunohistochemistry (E). **F.** PBX3 and G6PD mRNA expression levels in clinical human CRC and corresponding adjacent tissues, as analyzed using qRT-PCR (n = 20). **G-H.** PBX3 and G6PD protein expression levels in clinical human CRC (T) and corresponding normal adjacent tissues (N), as examined using western blotting (G) and immunohistochemistry staining (H). Cells transfected with shCon and pcCon were used as control. β-actin was used for qRT-PCR normalization and as the western blotting loading control. Scale bars: 20 μm Quantification data are shown as mean ± SD. pcEF9-Puro, **P < 0.01.

Next, to examine whether PBX3/G6PD axis affected tumorigenic potential, we established a *PBX3*-knocked down, *G6PD* overexpressed HCT116^p53null^ stable cell line and performed xenograft experiments** (Supplementary Figure S8)**. As shown in **Figure 6A**, knocking down *PBX3* significantly slowed down the growth of the xenografted tumors formed by HCT116^p53null^ cells, whereas *G6PD* overexpression prevented this suppressive effect, thus restoring the tumor growth rate as well as the size and weight of the generated tumors** (Figure 6B, C)**. The western blotting and immunohistochemistry results showed that G6PD expression was suppressed in the xenografted tumors formed by *PBX3*-knocked down HCT116^p53null^ cells **(Figure 6D, E)**.

Finally, we analyzed PBX3 and G6PD expression in clinical samples obtained from patients with CRC. The expression of both PBX3 and G6PD mRNA in tumor lesions increased significantly compared to adjacent tissues **(Figure 6F)**. The western blotting and immunohistochemistry results further confirmed the positive correlation between PBX3 and G6PD expression** (Figure 6G, H)**.

**Figure 7:**
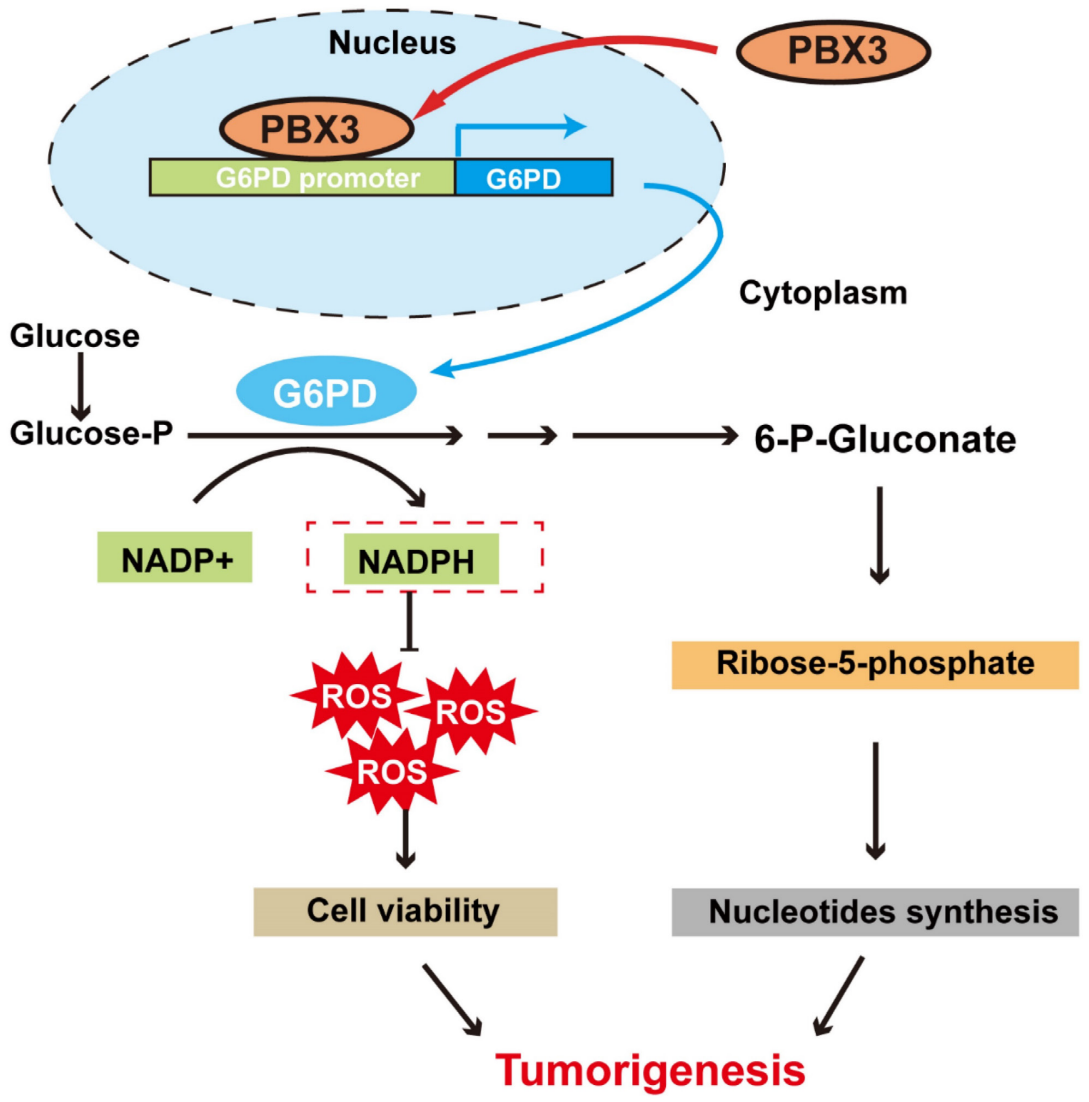
** Schematic diagram showing the PBX3/G6PD axis regulation on tumor cells PPP.** PBX3 binds directly to the *G6PD* promoter and promotes its transcription, leading to enhanced PPP and subsequently, tumorigenesis.

In summary, our study indicates that PBX3 stimulates PPP in tumor cells by directly activating the transcription of its rate-limiting enzyme, *G6PD*. Furthermore, PBX3-mediated PPP activation is crucial for its oncogenic function, most plausibly by increasing the antioxidant potential of the tumor cells and by providing them with building blocks for macromolecule biosynthesis **(Figure 7)**.

## Discussion

Aerobic glycolysis is a central metabolic feature of tumor cells that convert glucose into intermediate products favorable for tumor development 9, 36. Moreover, increasing evidences show that metabolic reprogramming plays critical roles in a range of different processes in cell proliferation and cell cycle progression, particularly in tumor cells 37, 38. Metabolic reprogramming drives malignant development as it provides several benefits to tumor cells, including macromolecular biosynthesis and energy. Furthermore, metabolic reprogramming could produce reductants to protect tumor cells from damage caused by excessive ROS; thus promoting tumor cells adaptation to their microenvironment 39-41. Our present study reveals that PBX3 is essential for glucose metabolism in tumor cells, specifically, we identify PBX3 as a novel transcriptional regulator of *G6PD* that binds directly to the *G6PD* promoter and enhances its activity. This leads to increased PPP flux, providing the cells with ROS scavengers and precursors of biomacromolecules which subsequently promotes tumorigenic potential.

Previous studies have shown that PBX3 is upregulated in multiple tumors, including colon, liver, gastric, and cervical cancers, as well as myeloma and glioma 18, 21, 24, 42-44. PBX3 could inhibit tumor cell apoptosis by activating the Raf1/MAPK1 pathway 43 and by suppressing tumor suppressor miRNAs, including miR-302, miR-129-5p, and miR-495 34-36. Furthermore, it could also activate the transcription of tumor-initiating cells-related genes, including calcium voltage-gated channel auxiliary subunit alpha 2 delta 1 (CACNA2D1), epithelial cell adhesion molecule (EpCAM), SRY-box transcription factor 2 (SOX2), and notch receptor 3 (Notch3), thereby promoting the self-renewal ability of tumor-initiating cells 21. Moreover, our previous study demonstrated that PBX3 regulates cell cycle progression and tumor growth by regulating the p53/p21 axis 24. Our current findings show that PPP activation is crucial for PBX3-mediated tumorigenesis, linking PBX3 with tumor cell glucose metabolic reprogramming, thus further confirming its function as an oncogene. It is noteworthy that overexpressing *G6PD* did not fully restore the tumorigenic potential suppressed by *PBX3* knockdown. Furthermore, although the effects were less significant compared to its regulation on G6PD, PBX3 also affects the levels of other factors involved in glucose metabolism, including glucose transporter 1 (GLUT1), which regulates the uptake of glucose into cells 45, as well as PGM1, PKM2, PDK1, and LDHA, which are involved in glycolysis 46. Thus, although further investigations are needed, PBX3 regulation in tumor metabolic reprogramming, and subsequently, tumorigenesis, might involve multiple pathways. Nevertheless, our current finding is the first that shows a crucial role of PBX3 in regulating tumor cell metabolic reprogramming through its regulation of G6PD.

G6PD upregulation occurs in various tumors and is strongly correlated with poor prognosis 47, 48. Previous studies have revealed that yin yang 1 (YY1) and TAp73 activate the transcriptional activity of *G6PD* 27, 49, while tumor suppressor gene p53 could inactivate its enzymatic activity 11. While whether PBX3 interacts with other factors in regulating *G6PD* transcriptional activity, or whether it could also regulate *G6PD* transcriptional activity through epigenetic regulation needs further investigation, our current study not only unravel a new mechanism regarding the oncogenic activity of PBX3, but also reveals a novel mechanism of G6PD/PPP activation in tumor cells. As an enzyme that catalyzes the first step of PPP, increased G6PD expression and enzymatic activity lead to enhanced PPP flux, thereby providing energy and precursors for macromolecule biosynthesis and subsequently driving tumor progression 50-54. Furthermore, enhanced PPP activity is also crucial for maintaining tumor cell redox homeostasis, which is critical for their proliferation and survival. The rapid proliferation of tumor cells leads to increased cellular ROS, thereby triggering oxidative stress. Previous studies have shown that although excessive ROS may damage or even kill tumor cells, tumor cells are more tolerant to ROS compared to normal cells, and a certain level of ROS could even benefit tumor progression 55-59. Besides fulfilling energy demands, increasing NADPH through PPP activation is assumed to be a strategy of tumor cells to address this problem. Moreover, as many DNA damage-based antitumor drugs trigger tumor cell apoptosis by inducing excessive ROS, increased PPP activity that elevates NADPH has attracted attention as a potential target to overcome tumor cell drug resistance 32. Together, these results highlight the importance of PPP in driving tumor progression and drug resistance. Accordingly, PPP is a potential target for an antitumor therapeutic strategy. Our present study unravels the novel function of PBX3 as a positive regulator of PPP. Inhibiting PBX3 could suppress PPP flux, thereby promoting tumor cell antioxidant defenses and proliferation, and subsequently, tumorigenic potential. Thus, PBX3 might be a potential target for antitumor therapeutic strategy targeting PPP.

In summary, we identified PBX3 as a novel regulator of tumor cell glucose metabolic reprogramming. Our results not only provide new insights regarding the regulation of tumor metabolic reprogramming but also a new perspective regarding the molecular mechanism underlying the oncogenic role of PBX3. Furthermore, PBX3 is a potential target for an antitumor therapeutic strategy.

## Supplementary Material

Supplementary materials and methods, figures and tables.
