# Supplementary material for: PBX3 promotes pentose phosphate pathway and colorectal cancer progression by enhancing G6PD expression
Source: Int J Biol Sci. 2023 Aug 28;19(14):4525–38. doi: 10.7150/ijbs.86279 (PMC10535713; doi:10.7150/ijbs.86279)
Supplement: Supplementary file 1 — Supplementary materials and methods, figures and tables. [file ijbsv19p4525s1.pdf]

Supplementary Materials for

PBX3 promotes pentose phosphate pathway and colorectal cancer progression by enhancing G6PD expression

Xinxin Luo, Mankun Wei, Wenfang Li, Hezhao Zhao, Vivi Kasim*, Shourong Wu*

*E-mail: shourongwu@cqu.edu.cn (S.W.)

vivikasim@cqu.edu.cn (V.K.)

This PDF file includes:

Supplementary Materials and Methods

Figure S1. Effect of PBX3 on glucose consumption and lactate production levels.

Figure S2. PBX3 positively regulates G6PD mRNA expression level.

Figure S3. Effect of PBX3 on intracellular NADPH and $NADP^+$ levels.

Figure S4. Effect of *PBX3* overexpression on tumor cells lipid accumulation.

Figure S5. G6PD is crucial for PBX3 regulation on NADPH and $NADP^+$ levels.

Figure S6. PBX3 regulates G6PD mRNA expression level in a p53-independent manner.

Figure S7. PBX3 enhances intracellular NADPH and $NADP^+$ levels in a p53-independent manner.

Figure S8. Establishment of *PBX3*-silenced, *G6PD*-overexpressed $HCT116^{p53null}$ stable cell line.

Table S1. Primer pairs used for qRT-PCR.

Table S2. Antibodies used for western blotting, ChIP assay, and immunohistochemistry.

Supplementary Materials and Methods

RNA extraction and qRT-PCR

Total RNA was extracted using Trizol (Invitrogen Life Technologies, Carlsbad, CA) according to the manufacturer's instructions. Total RNA sample (1 μg) was reverse-transcribed into cDNA using the PrimeScript Reagent Kit with gDNA Eraser (Takara Bio, Dalian, China), then qRT-PCR was performed with SYBR Premix Ex Taq (Takara Bio) to assess mRNA expression levels. Primer sequences for qRT-PCR are shown in Supplementary Table S1. β-actin was used to normalize sample amplification. The results are shown as relative to the expression level in the corresponding controls, which are assumed as 1.

Western blotting

Cells were collected and lysed with RIPA lysis buffer supplemented with a protease inhibitor and phosphatase inhibitor cocktail (complete cocktail; Roche Applied Science, Mannheim, Germany). An equal amount of samples (20 μg) were subjected to electrophoresis on sodium dodecyl sulfide polyacrylamide gels and transferred to polyvinylidene (PVDF) fluoride membranes with a pore size of 0.45 μm (Millipore, Billerica, MA). The membrane was then incubated with primary antibodies followed by secondary antibodies. Antibodies used are listed in Supplementary Table S2, and immunoblotting with anti-β-actin antibody was conducted to ensure equal protein loading. The signals were detected using the SuperSignal West Femto Maximum Sensitivity Substrate detection system (Thermo Scientific, Waltham, MA).

Quantification was performed using Quantity One, and the result was normalized using β-actin.

Apoptotic rate analysis

Cells were prepared as described above. Twenty-four hours after being re-seeded in 6-well plates, cells were collected and stained with Annexin V/PI using Apoptosis Assay Kit (Biosharp, Hefei, China). The apoptotic rate was determined using flow cytometry.

Nile red staining

Cells were fixed using 4% paraformaldehyde and stained with 0.01 mg/ml Nile red (Sigma-Aldrich, St. Louis, MO) for 20 min. Nuclei were stained with DAPI. Images were taken using Olympus IX7I (Olympus, Tokyo, Japan). Quantitative analysis was performed using ImageJ (NIH, Bethesda, MD). Fluorescence intensities were normalized by the corresponding cell number.

5-ethynyl-2′-deoxyuridine (EdU) incorporation assay

Cells were prepared as described above. Twenty-four hours after being re-seeded in 24-well plates, cells were subjected to EdU incorporation assay using BeyoClick™ EdU Cell Proliferation Kit with Alexa Flour 488 (Beyotime Biotechnology, Shanghai, China) according to the manufacturer’s instruction. Hoechst was used to stain the nuclei. Images were obtained using Olympus IX73 (Olympus). The ratio of *de novo*

DNA synthesis was calculated as the ratio of EdU-positive cells to Hoechst-positive cells.

Cell viability assay

Cells were prepared as described above and re-seeded in 96-well plates at a density of 5×10^3 cells/well. Cell numbers were counted at indicated time-points using a colorimetric assay with 3-(4,5-dimethylthiazol-2-yl)-5-(3-carboxymethoxyphenyl)-2-(4-sulfophenyl)-2H-tetrazolium (Promega, Madison, WI) in accordance with the manufacturer's instructions.

For the experiment with nucleosides, cells were re-seeded in 96-well plates at a density of 5×10^3 cells/well and cultured with medium containing 200 μM (final concentration) mixture of 4 ribonucleosides (adenosine, uridine, cytidine, and guanosine) and 4 deoxyribonucleosides (deoxyadenosine, deoxyguanosine, deoxythymidine, and deoxycytidine) in the presence or absence of ROS scavenger N-acetyl-L-cysteine (final concentration: 2 μM). Cell numbers were counted as described above three days after the cells were re-seeded.

Colony formation assay

Cells were prepared as described above, re-seeded into 6-well plates at a density of 300 cells/well, and cultured for 14 days. Cells were then fixed with 4% paraformaldehyde and stained with methylene blue. Quantification was then performed by counting the number of colonies formed.

Immunohistochemistry and hematoxylin-eosin (H&E) staining

Fresh human CRC tissue, normal adjacent tissue, and xenograft tumors were fixed overnight with 4% paraformaldehyde, embedded in paraffin, and sectioned to a thickness of 4 μm using cryostat. Sections were then dewaxed using xylene, rehydrated, and subjected to immunohistochemical staining. Briefly, the tissue sections were incubated with primary antibodies for 1 h, followed by incubation with corresponding secondary antibodies conjugated with horse-radish peroxidase. Visualization was performed using a DAB Kit (DAKO, Beijing, China) under a microscope. The nuclei were then counterstained with hematoxylin (Beyotime Biotechnology), followed by dehydration and coverslip mounting. The antibodies used were listed in Supplementary Table S2. Images were taken using Pannoramic Midi (3DHistech, Budapest, Hungary).

For hematoxylin-eosin (H&E) staining, paraffin sections from human colon cancer tissues, normal adjacent tissues, and mice subcutaneous tumors generated in xenograft experiment (4 μm thickness) were fixed in 10% formalin and washed with 60% propylene glycerol. The samples were stained with 0.5% hematoxylin-eosin (Sangon Bio, Shanghai, China) for 3 min followed by dehydration and coverslip mounting. Images were taken using Pannoramic Midi.

Dual luciferase reporter assay

Cells were co-transfected with indicated vectors, reporter vector bringing the firefly luciferase, and *Renilla* luciferase expression vector pRL-SV40 (Promega). After 24 h, luciferase activities were analyzed using the Dual Luciferase Reporter Assay

(Promega). The activities of the firefly luciferase reporters were normalized using those of *Renilla* luciferase.

Chromatin immunoprecipitation (ChIP) assay

Chromatin was immunoprecipitated using a ChIP Assay Kit (Beyotime Biotechnology) according to the manufacturer's instructions. In brief, after the cells were lysed, chromatins were then immunoprecipitated using protein A+G Agarose/salmon sperm DNA and anti-PBX3 antibody, anti-Histone H3 antibody, or normal rabbit IgG, and de-crosslinked for 4 h at 65 ℃. After being treated with 0.5 M EDTA, 1 M Tris (pH 6.5), and 20 mg/ml proteinase K, immunoprecipitated chromatin was then subjected to PCR by using PrimeSTAR Max (Takara Bio). The sequence of the forward primer used was 5'-TGT GAA TAT ACA TAC AAC AAA CCA T-3'; while that of the reverse primer was 5'-GCA CCA TCA CTC CCA GCT A-3'.

Fig. S1

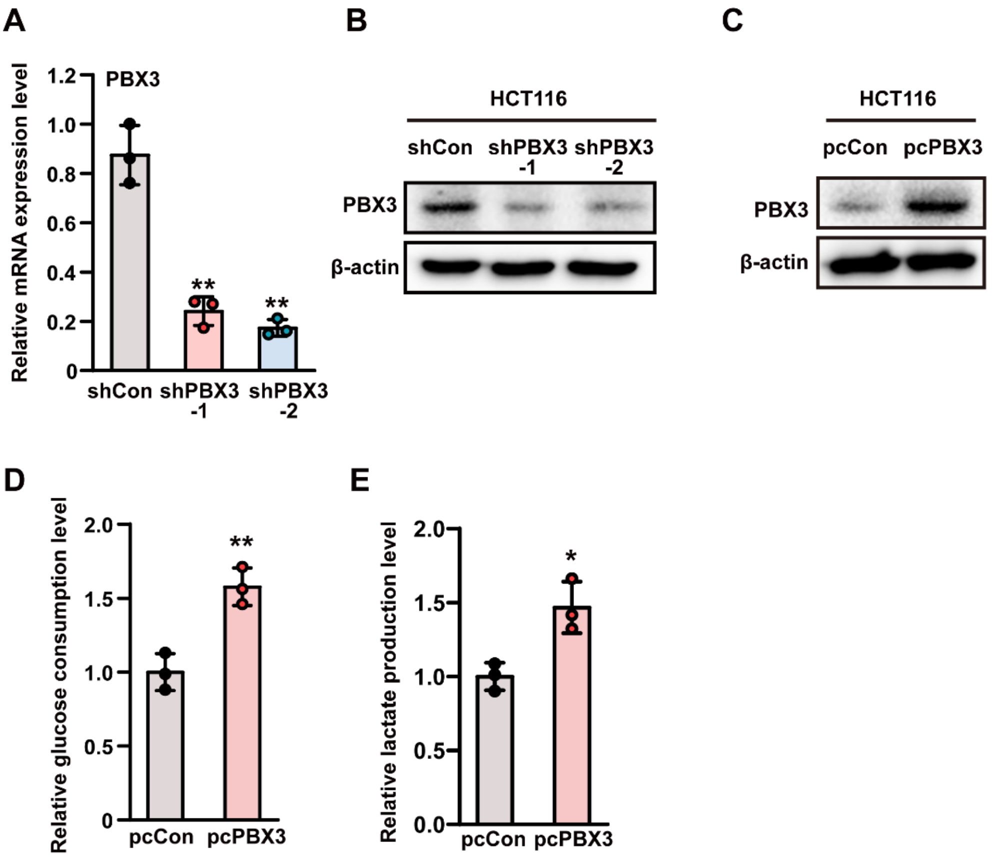

Fig. S1. Effect of PBX3 on glucose consumption and lactate production levels. A–B. PBX3 mRNA (A) and protein (B) expression levels in HCT116 cells transfected with two shRNA expression vectors targeting different sites of *PBX3*, as analyzed using qRT-PCR and western blotting, respectively. **C.** PBX3 protein expression level in HCT116 cells transfected with *PBX3* overexpression vector, as examined using western blotting. **D–E.** Glucose consumption (D) and lactate production (E) levels in *PBX3*-overexpressed HCT116 cells. Cells transfected with pcCon were used as control. β-actin was used for qRT-PCR normalization and as western blotting loading control. Total protein was used for normalizing glucose consumption and lactate production levels. Quantification data are shown as mean ± SD (n = 3). pcCon: pcEF9-Puro; $*P < 0.05$; $**P < 0.01$.

Fig. S2

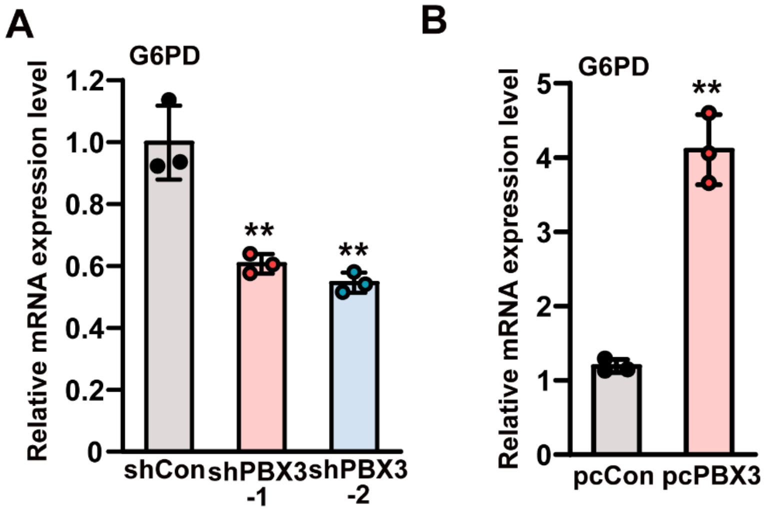

Figure S2. PBX3 positively regulates G6PD mRNA expression level. **A.** G6PD mRNA expression level in *PBX3*-knocked down HCT116 cells, as analyzed using qRT-PCR. **B.** G6PD mRNA expression level in *PBX3*-overexpressed HCT116 cells, as analyzed using qRT-PCR. β-actin was used for qRT-PCR normalization. Quantification data are shown as mean ± SD (n = 3). pcCon: pcEF9-Puro; ***P* < 0.01.

Fig. S3

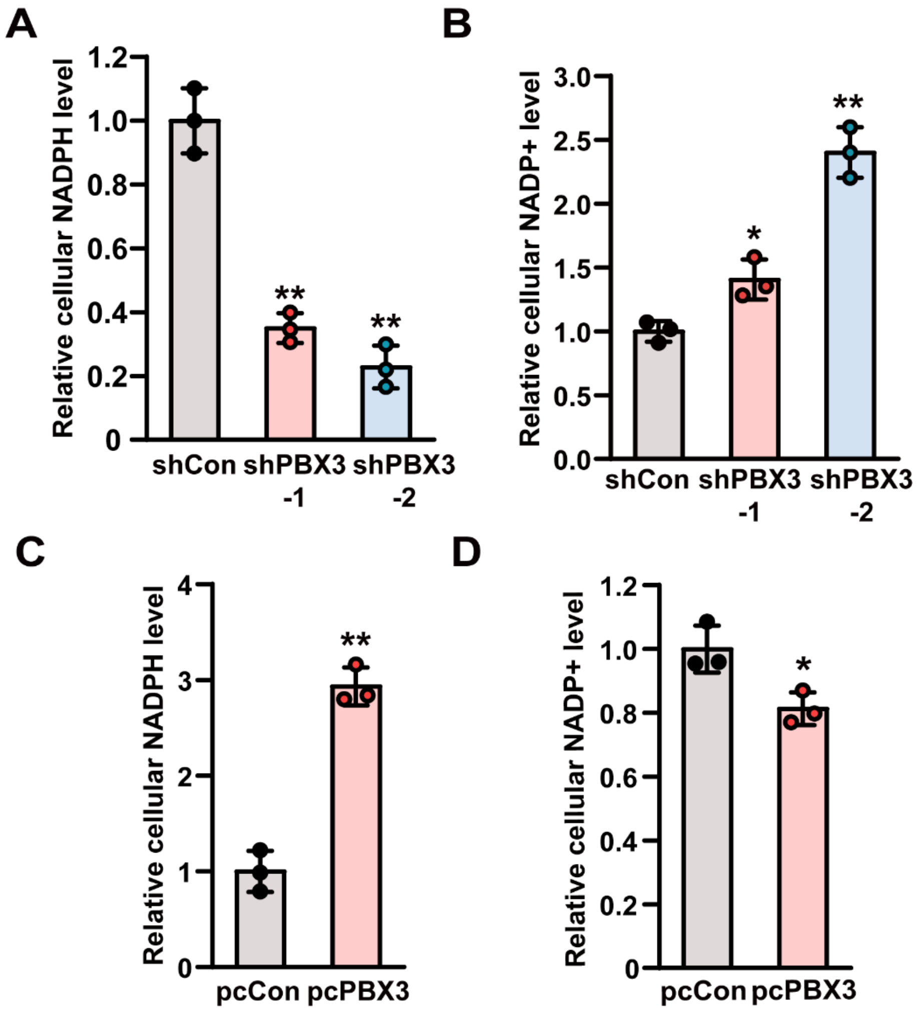

Figure S3. Effect of PBX3 on intracellular NADPH and NADP$^+$ levels. A–B. Intracellular NADPH (A) and NADP$^+$ (B) levels in *PBX3*-knocked down HCT116 cells. **C–D.** Intracellular NADPH (C) and NADP$^+$ (D) levels in *PBX3*-overexpressed HCT116 cells. Total protein was used for normalizing NADPH/NADP$^+$ level. Cells transfected with shCon or pcCon were used as control. Quantification data are shown as mean ± SD (n = 3). pcCon: pcEF9-Puro; **P* < 0.05; ***P* < 0.01.

Fig. S4

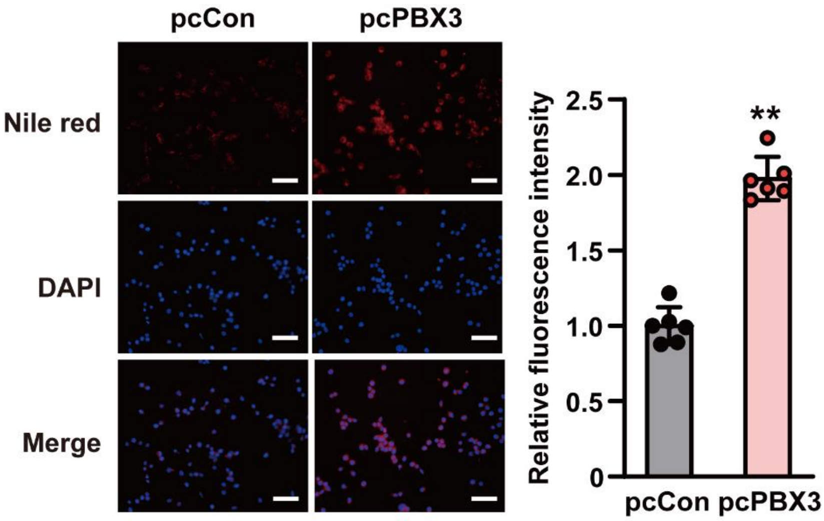

Figure S4. Effect of *PBX3* overexpression on tumor cells lipid accumulation. Lipid accumulation in *PBX3*-overexpressed HCT116 cells, as examined using Nile red staining. Representative images (left) and quantification results (right) are shown. Cells transfected with pcCon were used as control. Quantification data are shown as mean ± SD (n = 6). Scale bars: 200 µm. pcCon: pcEF9-Puro; ***P* < 0.01.

Fig. S5

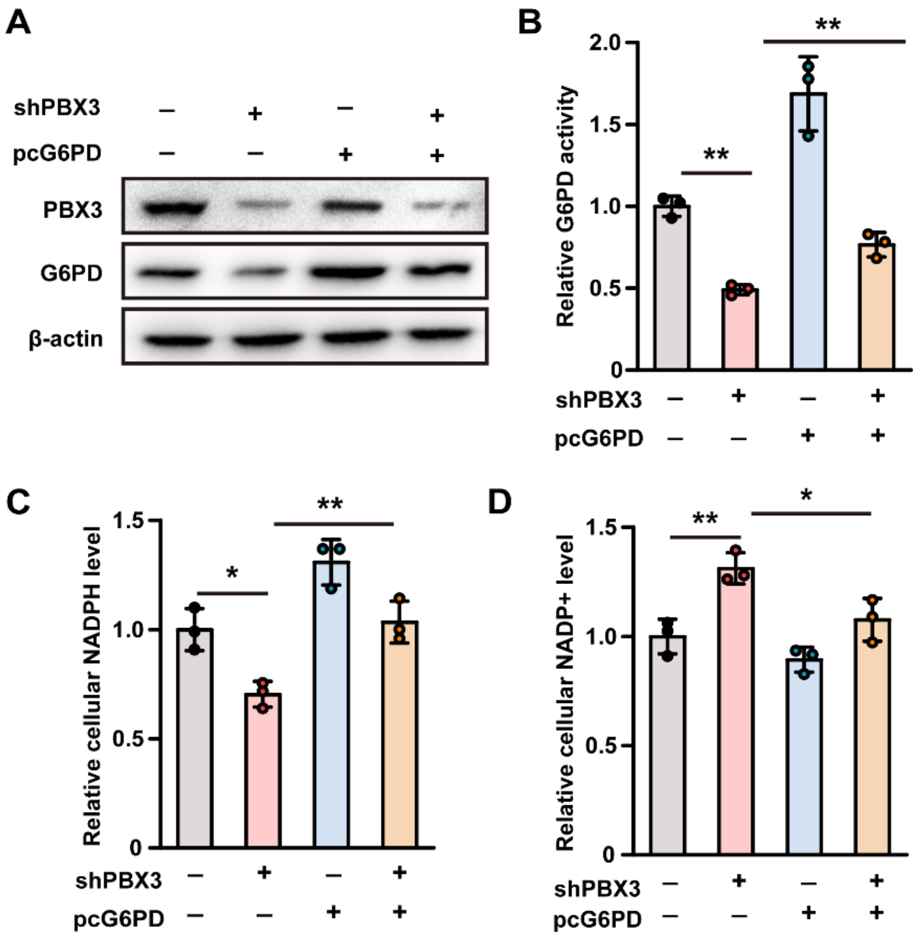

Figure S5. G6PD is crucial for PBX3 regulation on NADPH and NADP$^+$ levels. **A.** G6PD protein expression level in *PBX3*-knocked down, *G6PD*-overexpressed HCT116 cells, as determined by western blotting. **B**. G6PD enzymatic activity in *PBX3*-knocked down, *G6PD*-overexpressed HCT116 cells. **C–D.** Intracellular NADPH (C) and NADP$^+$ (D) levels in *PBX3*-knocked down, *G6PD*-overexpressed HCT116 cells. β-actin was used as western blotting loading control. Total protein was used for normalizing G6PD enzymatic activity, NADPH, and NADP$^+$ levels. Quantification data are shown as mean ± SD (n = 3). pcCon: pcEF9-Puro; $*P < 0.05$; $**P < 0.01$.

Fig. S6

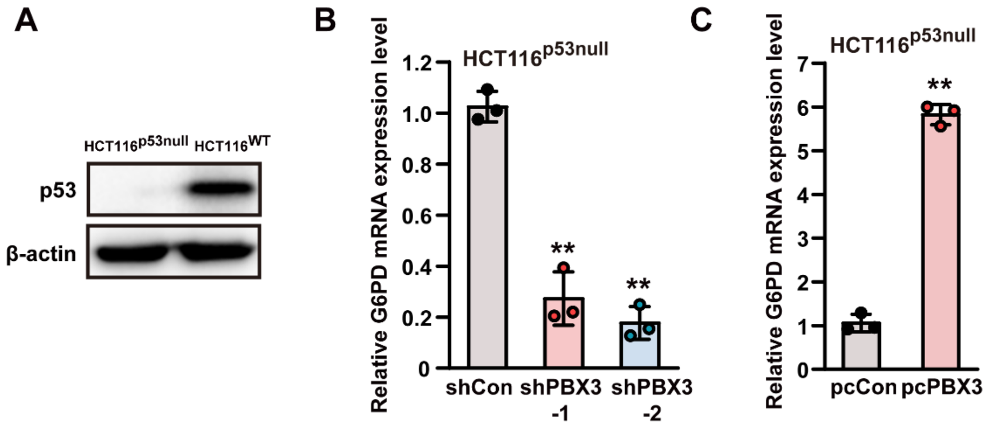

Figure S6. PBX3 regulates G6PD mRNA expression level in a p53-independent manner. A. p53 protein expression level in HCT116^{p53null} cells, as determined using western blotting. **B.** G6PD mRNA expression level in *PBX3*-knocked down HCT116^{p53null} cells, as analyzed using qRT-PCR. **C.** G6PD mRNA expression level in *PBX3*-overexpressed HCT116^{p53null} cells, as analyzed using qRT-PCR. β-actin was used for qRT-PCR normalization. Quantification data are shown as mean ± SD (n = 3). pcCon: pcEF9-Puro; $**P < 0.01$.

Fig. S7

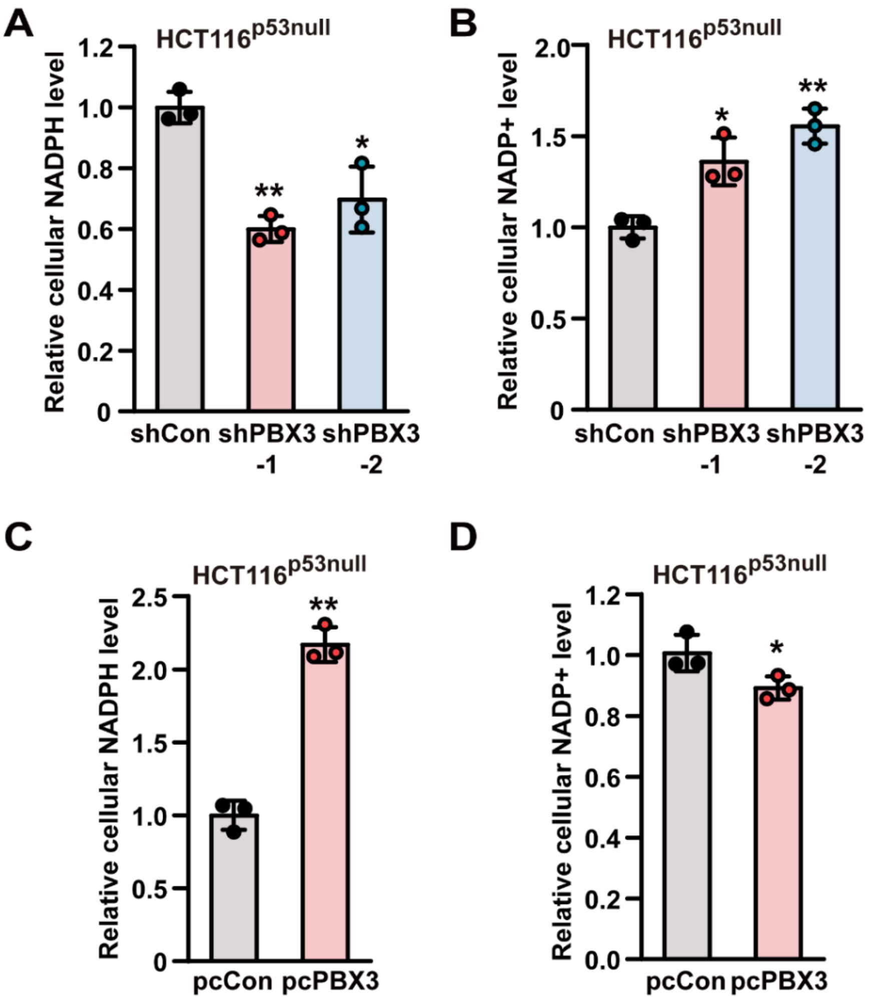

Figure S7. PBX3 enhances intracellular NADPH and NADP$^+$ levels in a p53-independent manner. A–B Intracellular NADPH (A) and NADP$^+$ (B) levels in *PBX3*-knocked down HCT116^{p53null} cells. **C–D** Intracellular NADPH (C) and NADP$^+$ (D) levels in *PBX3*-overexpressed HCT116^{p53null} cells. Total protein was used for normalizing NADPH/NADP$^+$ level. Cells transfected with shCon or pcCon were used as control. Quantification data are shown as mean ± SD (n = 3). pcCon: pcEF9-Puro; *$P < 0.05$, **$P < 0.01$.

Fig. S8

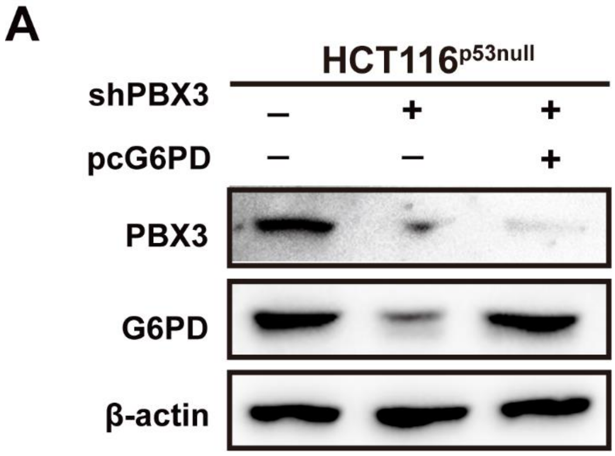

Figure S8. Establishment of *PBX3*-silenced, *G6PD*-overexpressed HCT116^{p53null} stable cell line. PBX3 and G6PD protein expression levels in *PBX3*-knocked down, *G6PD*-overexpressed HCT116^{p53null} cells, as determined using western blotting. β-actin was used as western blotting loading control. Cells transfected with shCon and pcCon were used as control; pcCon: pcEF9-Puro.

Supplementary Table S1. Primer pairs used for qRT-PCR

Genes	Refseq No.	Primer sequences (5'-3')	
		Forward	Reverse
PBX3	NM_001411009.1	TACAGAAGGCCCAGGAAGTG	AAAGCTGTAGATGGTGGGCT
G6PD	NM_000402.4	CGTGATGCAGAACCACCTAC	TGCATTTCAACACCTTGACC
GLUT1	NM_006516.4	ACCATTGGCTCCGGTATCG	GCTCGCTCCACCACAAACA
HK2	NM_000189.5	GATGACTTCCGCACAGAATTT	TCTCACCCAGGTACATTCCAC
TIGAR	NM_020375.3	CTCAAGACTTCGGGAAAGGA	GGTGTAAACACAGGGCACTCTT
PGK1	NM_000291.4	CTGTGGCTTCTGGCATACCT	CGAGTGACAGCCTCAGCATA
PGM1	NM_002633.3	CCGGTTCTACATGAAGGAGG	GAGGATTCCATTCTGTCCGA
PKM2	NM_001411081.1	ACGTGGATGATGGGCTTATT	CCAAGGAGCCACCATTTTC
PDK1	NM_001278549.2	CTGTGATACGGATCAGAAACCG	TCCACCAAACAATAAAGAGTGCT
LDHA	NM_005566.4	ACCCAGTTTCCACCATGATT	CCCAAA ATGCAAGGAACACT
SCO2	NM_005138.3	TCGTGCTTGGTCCACTGAC	TCAGCAGCAGCATGGATCT
FH	NM_000143.4	CGGTCAGGTCTGGGAGAAT	CCATGGTCATTGCTTCACAC
SDHC	NM_003001.5	TCAAACCGTCCTCTGTCTCC	AAGAGAGACCCCTGCACTCA
PFK2	NM_006212.2	GCTATGAAACCAAAACCCCA	TAACGATCAGAGTCGGGGAG
β-actin	NM_001101.5	CGAGCGCGGCTACAGCTT	TCCTTAATGTCACGCACGATTT

Supplementary Table S2. Antibodies used for western blotting, ChIP assay, and immunohistochemistry

Antibody	Product No.	Maker	Experiment	Dilution
Anti-PBX3	12571-1-AP	Proteintech	Western blotting<br>Immunohistochemistry<br>ChIP assay<br>Immunoprecipitation	1/1000<br>1/100<br>30 μg/mL cell lysate<br>25 μg/mL cell lysate
Anti-β-actin	60008-1-Ig	Proteintech	Western blotting	1/100000
Anti-G6PD	25413-1-AP	Proteintech	Western blotting<br>Immunohistochemistry	1/5000<br>1/500
Anti-p53	10442-1-AP	Proteintech	Western blotting	1/5000
Anti-Histone H3	17168-1-AP	Proteintech	ChIP assay	30 μg/mL cell lysate
Anti-Rabbit IgG	B900610	Proteintech	ChIP assay	30 μg/mL cell lysate
Goat Anti-Rabbit IgG	ZB2301	ZSGB-BIO	Western blotting	1/10000
Goat Anti-Mouse IgG	ZB2305	ZSGB-BIO	Western blotting	1/10000
